# Protective Effect of Silver Nanoparticles Against Cytosine Arabinoside Genotoxicity: An In Vivo Micronucleus Assay

**DOI:** 10.3390/ijerph21121689

**Published:** 2024-12-18

**Authors:** Idalia Yazmin Castañeda-Yslas, Olivia Torres-Bugarín, María Evarista Arellano-García, Balam Ruiz-Ruiz, Juan Carlos García-Ramos, Yanis Toledano-Magaña, Alexey Pestryakov, Nina Bogdanchikova

**Affiliations:** 1 Centro de Nanociencias y Nanotecnología, Universidad Nacional Autónoma de México, Ensenada 22860, Baja California, Mexico; icastaneda@ens.cnyn.unam.mx; 2 Medicina Interna II, Decanato Facultad de Medicina, Universidad Autónoma de Guadalajara, Zapopan 45129, Jalisco, Mexico; oliviatorres@hotmail.com; 3 Facultad de Ciencias, Universidad Autónoma de Baja California, Ensenada 22860, Baja California, Mexico; 4 Escuela de Ciencias de la Salud Unidad Valle Dorado, Universidad Autónoma de Baja California, Ensenada 22890, Baja California, Mexico; bruiz@uabc.edu.mx; 5 Instituto Tecnológico de Ensenada, Tecnológico Nacional de México, Ensenada 22780, Baja California, Mexico; jgarcia@ite.edu.mx (J.C.G.-R.); yanistoledano@gmail.com (Y.T.-M.); 6 Centro de Bachillerato Tecnológico Industrial y de Servicios No. 41” Belisario Domínguez”, Dirección General de Educación Tecnológica Industrial, Ensenada 22785, Baja California, Mexico; 7 Research School of Chemistry and Applied Biomedical Sciences, Tomsk Polytechnic University, Tomsk 634050, Russia; pestryakov2005@yandex.ru

**Keywords:** genoprotective effect, silver nanoparticles, cytosine arabinoside, in vivo assay, micronucleus assay&gt

## Abstract

Cancer treatments have harmful side effects, including genotoxic ones. Our previous research discovered that a specific silver nanoparticle (AgNPs) formulation could reduce the genotoxic effects of an alkylating agent, cyclophosphamide. This study aims to evaluate if this protective effect is observed against an antimetabolite anticancer agent, cytosine arabinoside (Ara-C). An erythrocyte micronucleus assay was conducted on BALB/c mice. A most significant effect was observed after the application scheme, including three doses of Ara-C and three subsequent doses of AgNPs, resulting in a 3.7 and 2.0-fold decrease in the frequency of micronucleated reticulocytes and accumulated erythrocytes, respectively. Current and previous studies reveal that AgNPs could be used as a genoprotector against the genotoxic damage produced by the currently used antineoplastic antimetabolites and alkylating agents. It was revealed that AgNPs could be considered a new class of promising synthetic antineoplastic genoprotectants along with the known class of derivatives from natural sources.

## 1. Introduction

Adverse effects of antineoplastic drugs have been described for decades, leading different research groups to develop new drugs for an efficient and safe treatment of cancer patients [1]. However, the antineoplastic agents currently used still present adverse severe effects, especially potential mutagenic and genotoxic effects [2]. Furthermore, the consequences of drug toxicity have been reported in health personnel specialized in oncology as well as in patients under treatment [3]. Therefore, it is urgent to find a more efficient antineoplastic treatment with high selectivity to cancer cells, causing less genotoxic damage to healthy cells and less long-term environmental damage.

The antineoplastic agents currently used in cancer treatment are classified according to their chemical structure and mechanism of action: antimetabolites, alkylating agents, monoclonal antibodies, natural products, and hormones [4,5,6]. These drugs have proven to be effective against cancer cells but also lead to numerous adverse reactions. Some authors point out that very few clinical trials have been conducted on antineoplastic agents compared to drugs for treating other diseases due to the regulations applicable in some countries [4]. This often results in shorter administrations and lower doses for patients. Consequently, limited information is available regarding the potential side effects when the drugs are marketed.

Ara-C is a widely prescribed antineoplastic drug for treating acute myeloid leukemia [7]. It is within the group of antimetabolites in the subgroup of pyrimidine equivalents. This group is fundamental in antineoplastic treatment because of its broad-spectrum activity [8]. Cytarabine, Ara-C, or cytosine arabinose are synonyms of these antineoplastics [9]. Ara-C is an analog of histidine and deoxycytidine. Its action mechanism is the inhibition of DNA polymerase directly on acid kinases, thus exerting a cytotoxic effect on nucleic acids [8]. Ara-C is used for the blast phase of chronic myeloid leukemia, acute myeloid leukemia, meningeal leukemia, non-Hodgkin’s lymphomas, and erythroleukemia treatment because it acts on rapidly proliferating cells. The adverse effects reported under a therapeutic regimen specifically for myeloid leukemia are cirrhosis, veno-occlusive disease, hepatomegaly, myelosuppression, gastrointestinal toxicity, central nervous system, skin, and eye toxicity [8,9,10].

Our group revealed that a formulation of AgNPs has significant antiproliferative activity and is highly selective against cancer tumor cells [11,12], without genotoxic effects [11], as well as mitigating adverse effects on the environment [13]. This formulation can decrease the genotoxic effect of cyclophosphamide (CP), which is the first-line antineoplastic drug for some cancer types [14]. The results of that work show the need to clarify whether this effect is observed only for cyclophosphamide or other first-line antineoplastic drugs with different mechanisms of action, such as Ara-C [15], and increase the number of application schemes for better results perception. If this effect could also be observed for Ara-C, the treatment of cancer with this formulation in the future could allow the treatment of patients without or with minor side effects caused by the genotoxicity of this current first-line drug.

It is important to emphasize that AgNPs have been extensively studied in veterinary medicine, human medicine, agriculture, and aquaculture [16,17,18]. For its future application as a genoprotectant against the genotoxicity of antineoplastic drugs, toxicological studies are of great importance. Several works reported the results of toxicological studies of this AgNPs formulation: the cytotoxicity and genotoxicity of human lymphocytes [19], the hemolysis of erythrocytes from healthy and diabetic donors [20], environmental toxicity in the Allium cepa model [13], toxicokinetic and toxicodynamic [21], hematological parameters, and the No Observed Adverse Effect Level (NOAEL) [22], among others. In addition, the AgNPs formulation was studied in human pilot clinical trials for the treatment of diabetic foot ulcers [18], COVID-19 prophylaxis in medical personnel [23], and acute respiratory diseases [24]. All these studies demonstrated that AgNPs exhibit very low toxicity at therapeutic doses. Therefore, this work aimed to determine if AgNPs that already have shown genoprotective properties against the first-line anticancer drug cyclophosphamide [14] have properties like those of another oncological drug (cytosine arabinoside).

## 2. Materials and Methods

### 2.1. AgNPs

The Argovit™ AgNPs formulation used in this study was generously provided by the Scientific and Production Center Vector-Vita (Novosibirsk, Russia). The physicochemical properties of Argovit™ AgNPs have been previously characterized and reported by our group [25]. Briefly, Argovit™ AgNPs are spherical nanoparticles with an average size of 35 nm and a range between 1 and 90 nm. These nanoparticles are coated with polyvinylpyrrolidone (PVP) (18% *w*/*w*), which serves as a stabilizer, and are dissolved in distilled water at a concentration of 1.2% (*w*/*w*) of metallic silver. So, the concentration of AgNPs (metallic Ag with stabilizer) in water was 20%. The surface plasmon resonance of these nanoparticles is observed at 420 nm.

The hydrodynamic diameter of Argovit™ AgNPs is 70 nm, and their zeta potential is −15 mV. While this zeta potential is below the commonly accepted threshold of ±30 mV for colloidal stability, the stability of Argovit™ AgNPs is maintained through steric stabilization provided by the PVP coating. This unique combination of properties ensures consistent dispersion and stability of the nanoparticles in solution, making them suitable for biological applications and further experimental studies [26].

### 2.2. Animals

The study was conducted on male *Mus musculus* mice of the BALB/c strain of 5 to 6 weeks of age and a mean weight of 20.46 ± 2.96 g. The experiments included a total of 49 BALB/c mice. The animals were obtained from the Centro de Investigación Biomédica de Occidente (CIBO) at the Instituto Mexicano del Seguro Social (IMSS) in Guadalajara, Jalisco, México. The animals were placed inside polycarbonate cages bedded with sawdust powder, which were acclimated to the laboratory conditions for two weeks before the treatment application, and fed ad libitum with standard rodent pellets and water. At the beginning of every day of the experiment, the animals were weighed to update the dosage of each treatment [27].

### 2.3. Detailed Explanation of the Design of Experimental Groups

**Figure 1:**
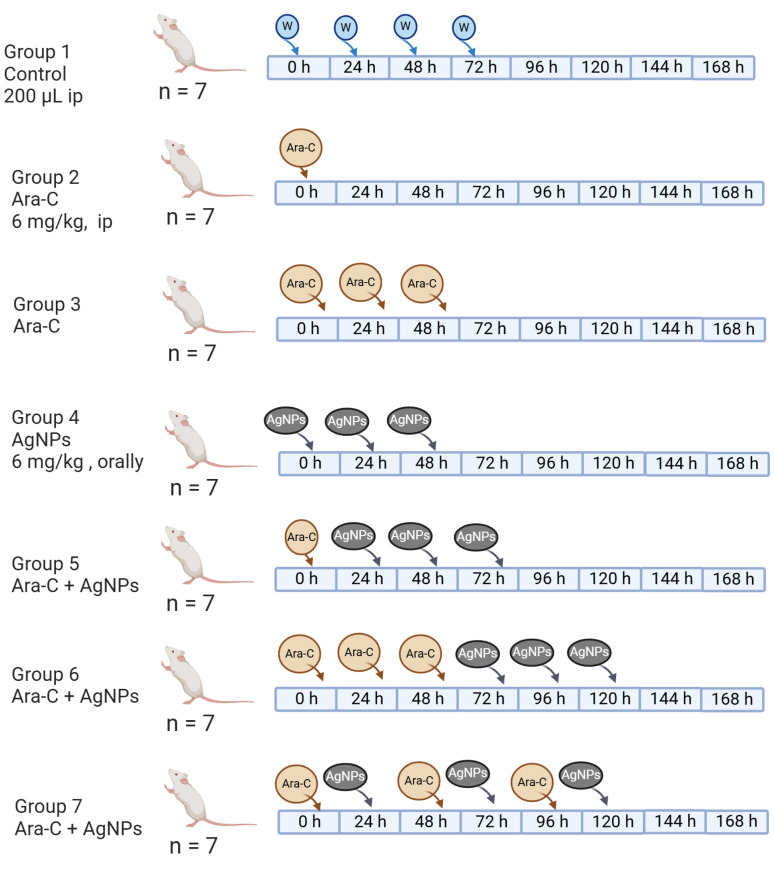
Administration schemes with Ara-C. Group 1—Control (0, 24, 48 h), group 2—Ara-C (0 h), group 3—Ara-C (0, 24, 48 h), group 4—AgNPs (0, 24, 48 h), group 5—Ara-C (0 h) + AgNPs (24, 48, 72 h), group 6—Ara-C (0, 24, 48 h) + AgNPs (72, 96, 120 h) and group 7—Ara-C (0, 48, 96 h) + AgNPs (24, 72, 120 h).

The experimental design included seven groups of BALB/c mice, each specifically designed to investigate the effects of AgNPs, Ara-C and their combination of cytotoxicity and genotoxicity (Figure 1).

We established the dosage of Ara-C at 6 mg/kg because previous studies showed that this amount is effective for repeated intraperitoneal administration in BALB/c mice [28]. We set the dosage of silver nanoparticles (AgNPs) based on earlier research that showed they can effectively manage the Rift Valley fever virus [25]. We prepared dilutions of the original AgNPs formulation (water suspension) at 1:5000 and 1:10,000 dilution for oral administration.

– Group 1 was the control, which only received 200 µL of water orally, which served as a baseline.
– Group 2 received 6 mg/kg of Ara-C (Sigma-Aldrich, St. Louis, MO, USA; CAS No. 147-94-4) intraperitoneally (IP) to assess its acute cytotoxic and genotoxic effects.
– Group 3 received IP 6 mg/kg of Ara-C for three consecutive days to evaluate its chronic impact.
– Group 4 received 6 mg/kg of AgNPs (Argovit^TM^) orally for three consecutive days, equivalent to 6 mg/kg metallic silver, which was used to determine their independent cytotoxic or genotoxic potential.
– Group 5 received a single IP dose of Ara/C at 6 mg/kg and three consecutive days of AgNPs at the exact dosage orally to evaluate the protective effects of AgNPs.
– Group 6 received three consecutive IP doses of Ara-C at 6 mg/kg each and three oral doses of AgNPs, mimicking both single and repeated exposure scenarios.
– Group 7 received an alternative dosing schedule, starting with 6 mg/kg of Ara-C on day one and 6 mg/kg of AgNPs on day two, for six consecutive days, to investigate whether alternating treatments influenced the genoprotective effects.

For this study, the nanoparticles were administered as an aqueous suspension. The stock preparation was diluted with distilled water, and doses were adjusted daily based on the weight of each mouse to ensure accurate dosing [29].

### 2.4. Cytotoxicity and Genotoxicity

In vivo experiments to determine the cytotoxicity and genotoxicity of Ara-C and AgNPs were carried out according to the Organization for Economic Cooperation and Development (OECD) procedure with some modifications [30].

During the treatment, a drop of blood was taken from each mouse tail tip before and after each administration every 24 h up to 168 h [31]. Two smears were prepared per organism on a slide, which was labeled beforehand. The samples were allowed to dry in the open air for 10 min and fixed in ethanol (80%) for another 10 min. After fixing, the samples were dried in the open air again. Once dry, they were stained with acridine orange (prepared by adding 0.05 g of acridine orange in 250 mL of phosphate buffer (PBS) solution with a final pH of 6.8). The slides were placed on grids, stained for one minute, and then rinsed on the PBS solution to remove excess dye [28].

All slides were coded prior to microscopic analysis. The same researcher evaluated the samples, manually scoring them using a Carl Zeiss^®^ epifluorescence microscope (Carl Zeiss, Oberkochen, Germany) equipped with a 100× oil-immersion objective. The frequency of micronucleate normochromatic erythrocytes (MNE) was assessed in a total of 10,000 total erythrocytes (TE). Additionally, the proportion of polychromatic erythrocytes (PCE) was counted in 1000 TE to evaluate a decrease in cellular division, which indicates cytotoxic effects. The micronucleated young erythrocytes (MNPCE) frequencies were also determined in 1000 PCE. Acridine orange staining resulted in PCE appearing red or orange, while normochromatic (mature) erythrocytes were dark green [31].

### 2.5. Ethical Considerations

The sacrifice was performed according to NOM-033-SAG/ZOO-2014 [32]. The project was approved under protocol #002/2021 by the Research Ethics Committee of the Facultad de Ciencias de la Salud, Valle de las Palmas.

### 2.6. Statistical Processing

The results of the PCE, MNPCE, and MNE readings were recorded in an Excel^®^ sheet for later normalization and graphic representation with the GraphPad10 software (version 10.4.1., GraphPad Software, Boston, MA, USA). The results are expressed based on the mean value and the standard error of the mean (SEM), which was determined by the quotient of the standard deviation over the square root of the number of replicates multiplied by the value of Z = 1.96 for 95% confidence. The effect of the different treatment schemes on the biomarkers of EPC cytotoxicity and MNPCE short-term and MNE long-term genotoxicity was determined based on an analysis of variance and multiple comparisons a posteriori by Tukey’s test.

## 3. Results

### 3.1. Animal Conditions

Throughout the seven-day experiment, the average weight of the mice varied based on the mean and standard error, as shown in Table 1. In Group 5, two mice died—one on the first day and another on the second day. Furthermore, in Group 4, two mice exhibited slight body bending for two days after treatment, which was accompanied by softened feces. These symptoms resolved after two days.

### 3.2. Cytotoxicity and Myelosuppression

**Figure 2:**
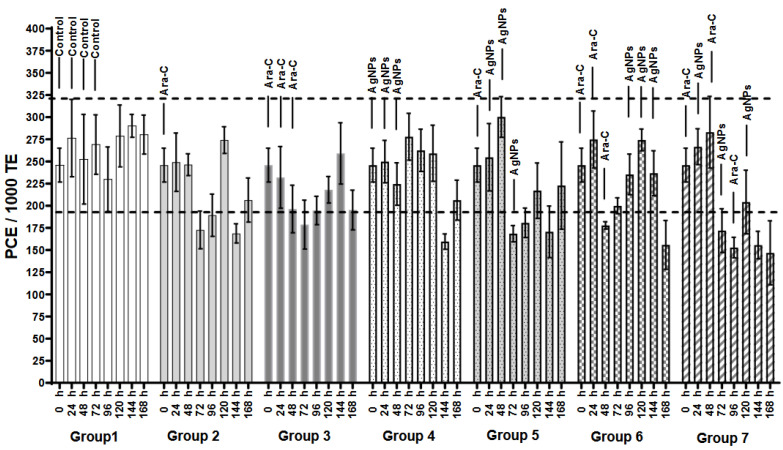
Frequency of polychromatic erythrocytes (PCE/1000 TE). The dotted lines indicate PCE variation in the control group. Bar height represents the mean, with error bars showing the standard error. The Supplementary Excel file contains ANOVA results (*p* < 0.05) and Tukey’s test.

Myelosuppression is a result of cytotoxicity. The PCE/1000 TE decrease is the biomarker of cytotoxicity (Figure 2) [33]. The negative control group presented PCE frequencies ranging from 190 to 320 PCE/1000 TE, while the two groups treated with Ara-C (Groups 2 and 3) showed lower frequencies (170-270 PCE/1000 TE), reflecting the cytotoxic effect after one and three consecutive days of antineoplastic administration (myelosuppression). However, the bone marrow recovered within 168 h, manifested as an increase in PCE levels back to average levels in these two treatments, without any signs of myelosuppression. Conversely, Group 4, administered with AgNPs for three consecutive days, exhibited myelosuppression (Figure 2). Nevertheless, the PCE production in Group 4 recovered 24 h later.

In Groups 5 and 7, using Ara-C and AgNPs together resulted in a noticeable decrease in PCE at 72 h. Group 5 received a single dose of Ara-C and three doses of AgNPs. At 168 h, an increase in PCE count was observed, indicating bone marrow recovery. In contrast, in Groups 6 and 7 that received Ara-C+ AgNPs (see Figure 2), the bone marrow showed slight recovery between 72 and 144 h. However, at 168 h, severe myelosuppression was observed in both groups.

### 3.3. Acute Genotoxicity

**Figure 3:**
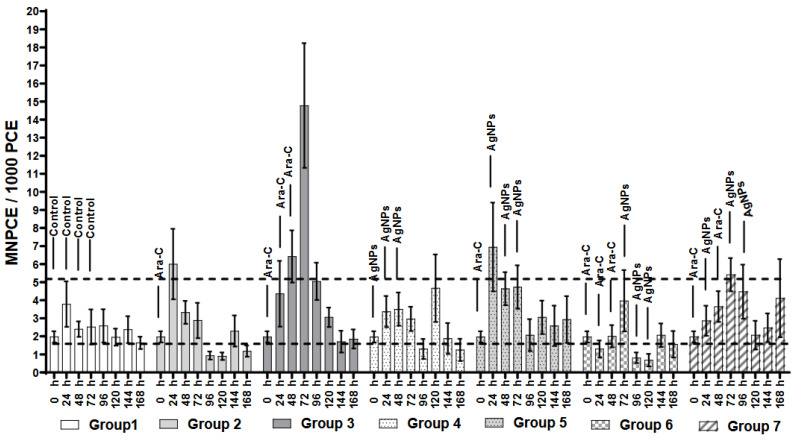
Frequency of micronucleated polychromatic erythrocytes (MNPCE/1000 PCE). The dotted lines indicate MNPCE variation in the control group. Bar height represents the mean, with error bars showing the standard error. Supplementary Figure S1 contains ANOVA results (*p* < 0.05) and Tukey’s test.

The frequency of MNPCE is evidence of acute genotoxicity (Figure 3). In the control group, this biomarker was maintained constant. In Group 2, a single dose of Ara-C led to a maximum increase in MNPCE of 5.9 ± 1.9 one day post-administration; however, levels returned to normal for the rest of the experiment. In contrast, administering three daily doses of Ara-C in Group 3 consistently elevated MNPCE over the three consecutive days following treatment. This approach reached a peak of 14.8 ± 3.4 at 72 h, which was 3.9 times higher than the maximum MNPCE recorded in the control group 2.5 ± 2.5 (*p* < 0.05). In Group 4, three daily doses of AgNPs did not cause acute DNA damage for most of the experiment [27]. The highest recorded damage level was 4.6 ± 1.8 at 120 h, but levels returned to normal in the final two days.

According to Figure 3, the combined treatments in Groups 5–7 produced interesting results. In Group 5, administering one dose of Ara-C followed by three doses of AgNPs increased MNPCE with the highest recorded at 24 h (6.947 ± 2.5). However, this increase was not significantly different (*p* > 0.05) compared to the single dose of Ara-C in Group 2 (5.9 ± 1.9). The other two combined treatments (Groups 6 and 7) did not exceed the control limits regarding MNPCE. The administering of one dose of Ara-C and three subsequent doses of AgNPs (Group 5) caused an increase in MNPCE with the highest recorded at 24 h (6.947 ± 2.5) for Group 5. However, there was no significant difference (*p* > 0.05) in the behavior of Group 5 compared to the single dose of Ara-C in Group 2 (5.9 ± 1.9). While Groups 6 and 7 exhibit similar profiles, Group 6 resulted in a lower MNPCE count than Group 7. In contrast. The MNPCE levels remained within the control group limits in both treatment combinations.

### 3.4. Chronic Genotoxicity

**Figure 4:**
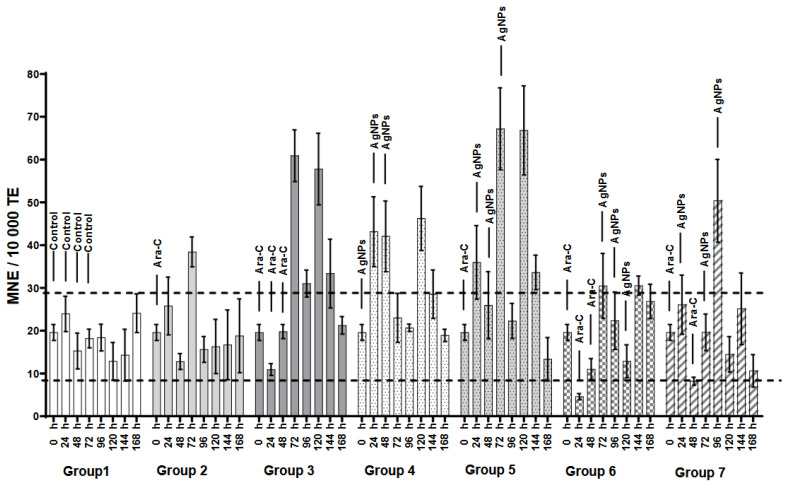
Frequency of micronucleated erythrocytes per 10,000 total erythrocytes (MNE/10,000 TE). The dotted lines indicate MNE variation in the control group. Bar height represents the mean, with error bars showing the standard error. The Supplementary Excel file contains ANOVA results (*p* < 0.05) and Tukey’s test.

Chronic genotoxicity is assessed using the MNE/10000 TE (Figure 4) [34], which tracks erythrocytes from the bloodstream to the red bone marrow. Most comparisons showed a significant difference (*p* < 0.05) between the control group and others. In Group 2, the MNE per 10,000 TE increased to 38 ± 3.5 MN at 72 h (*p* < 0.05) but declined afterward. In Group 3, the MNE values were 60.9 ± 6 at 72 h and 33.3± 8.0 at 144 h.

In Figure 4, Group 4 showed a significant increase in genotoxicity (*p* < 0.05) at 24 until 120 h, but Group 5 was until 144 h. Conversely, Group 6 did not show a significant increase in MNE/10,000 TE compared to the control group (Group 1). On the other hand, Group 7 had only one significant increase at 96 h.

Unexpectedly, in Group 6, the alternating treatment scheme in Group 7 effectively reduced the genotoxicity associated with Ara-C. While AgNPs alone increased genotoxicity in Group 4, Groups 6 and 7 exhibited lower genotoxic effects. Notably, the alternating treatment in Group 7 proved more effective in mitigating the genotoxicity from Ara-C compared to Group 3, which received only Ara-C, and Group 4, which administered three dos of AgNPs.

## 4. Discussion

The study examined, using the biomarkers PCE, MNPCE, and MNE, the protective effect of AgNPs against Ara-C, an antineoplastic drug, focusing on their cytotoxicity and genotoxicity in peripheral erythrocytes when administered alone and combined with silver nanoparticles. Ara-C causes acute cytotoxicity within a few hours, as the PCE analysis shows (Figure 2); however, this effect diminishes after 10 to 12 h because this compound is rapidly eliminated from circulation [35]. Still, with repeated doses, the effect prevails for longer, which is probably due to the cumulative impact on bone marrow [36].

The impact of AgNPs on bone marrow was not as enduring as that of Ara-C. When applied repeatedly, AgNPs did not induce immediate cytotoxicity, as evidenced by Figure 2. Compared to Ara-C, orally administering AgNPs at a 6 mg/kg dose results in a shorter duration when they remain in the bloodstream; the pharmacokinetic behavior of AgNPs could also explain this, since some analyses suggest that low to medium doses of AgNPs are more easily absorbed by the small intestine. In contrast, higher doses affect the kidneys through the bloodstream [37]. It is unclear how AgNPs change in structure and function as they traverse various tissues and organs, such as the mouth, esophagus, intestines, bloodstream, liver, and kidneys [38]. The toxic response to oral ingestion of AgNPs is multifaceted and not solely determined by the dosage [39]. Another explanation is that in the BALB/c model, the oral administration of AgNPs creates a bio-corona that alters their physicochemical and toxicological properties [40].

These findings emphasize the need for future studies to investigate the long-term effects of AgNPs on hematological parameters such as neutrophils and platelets [41,42]. This would provide a more comprehensive assessment of their impact on bone marrow function and overall hematological health. Such studies would also help clarify how AgNPs interact with specific tissues and whether their protective effects extend beyond the markers evaluated in this study [43,44]. Understanding these mechanisms will be essential to optimizing the use of AgNPs as genoprotectants and assessing their safety profile in more complex biological systems [1,2].

The analysis of MNPCE (Figure 3) shows that repeated doses of AgNPs can reduce the acute genotoxicity induced by Ara-C when erythrocytes are treated with alternating doses. Combining AgNPs with Ara-C decreases acute genotoxic damage by three- to four-fold. This contrasts when Ara-C is applied in similar doses and schedules, resulting in a significant increase in acute genotoxicity (MNPCE) (*p* < 0.05). This result suggests a genoprotective effect of AgNPs against the damage produced by the antineoplastic Ara-C. It correlates with a genoprotective effect of AgNPs against the damage produced by the antineoplastic cyclophosphamide [14].

Further studies, for example, using in vitro cytokinesis block assay of lymphocytes (L-CBMN) [11] are needed to understand the genoprotective mechanisms of AgNPs [45,46] and to elucidate, in addition to its cytotoxic effect, the cell death pathway that these Ara-C and AgNPs combinations induce, since the L-CBMN assay provides six biomarkers: three for cytotoxicity and three for genotoxicity [19]. The results of the present work coincide with the results of studies that report the genoprotective effect in vivo and in vitro of different compounds against such antineoplastic drugs as cyclophosphamide, arabinose, ifosfamide, and mitomycin, which allows us to propose that there may be several mechanisms associated with the interaction of antineoplastics with different formulations that act as a genoprotectant linked to the mechanism of apoptosis [47,48] via glutathione depletion [49].

In the examination of MNE, the accumulative genotoxicity can be revealed (Figure 4). Three doses of AgNPs have been found to effectively decrease the genotoxicity resulting from Ara-C both when used alone and in combination with AgNPs. Our findings indicate that compared to Ara-C, the cytogenetic damage induced by AgNPs is less severe (*p* < 0.05) when each was administered over three consecutive days. This is attributed to the lower levels of cytotoxicity and accumulative genotoxicity (MNE/10,000 TE) associated with AgNPs. The combination of Ara-C and AgNPs exhibits a genoprotective effect on MNPCE, which is similar to the effect observed in our previous research on cyclophosphamide [14]. However, the effect is more significant with Ara-C, resulting in a fourfold reduction in MNPCE and a twofold reduction in MNE compared to the twofold decrease in MNPCE and 1.32-fold decrease in MNE observed with cyclophosphamide [14].

The study did not test uncoated silver nanoparticles or PVP alone. However, a previous study by Ruiz-Ruiz et al. (2020), which used a similar experimental setup with human lymphocytes, demonstrated that the complete nanoparticle–PVP formulation was responsible for the observed protective effect. These findings suggest that the protective effects are likely due to the synergistic interaction between the silver nanoparticles and the PVP coating rather than either component alone [19].

The genoprotective effects of Ag, Se, and TiO_2_ nanoparticles in vitro have been published recently [50,51]. The research shows, for example, that plant extracts and Ag and Se nanoparticles coated with plant extracts exhibit a genoprotective effect in vitro [40,41,49,50]. However, TiO_2_ nanoparticles coated with an extract of *Sargassum polycystum* did not demonstrate any genoprotective effect [52]. Additionally, apigenin plant extract (C_15_H_10_O_5_) and AgNPs coated with *Ocimum Sanctumleaf* extract were discovered to reduce chromosomal aberrations in human peripheral blood caused by cyclophosphamide up to 4.5 and 4.1 times, separately [52]. Furthermore, apigenin plant extract and selenium nanoparticles coated with extract plants have been shown to lower genotoxic damage in human lymphocytes and erythrocytes by 3.2 and 3.1 times, respectively [50,53].

This study provides evidence of the genoprotective effects of AgNPs against Ara-C-induced genotoxicity in an in vivo mouse model. The results demonstrate significant reductions in MNE when AgNPs are administered following Ara-C treatment, yet the molecular mechanisms underlying these effects remain unexplored. Mechanistic insights, such as the expression of cyclin-dependent kinases (CDK1/2), critical cell cycle regulators, and DNA repair regulators, could provide valuable information to understand these findings further. CDK1/2 activity, often implicated in cellular responses to DNA damage, represents a promising target for future investigations to determine whether AgNPs modulate these pathways to exert their protective effects. For instance, studies have shown that CDK1/2 and other markers of cell cycle progression play essential roles in maintaining genomic stability and mitigating genotoxic damage [54] and arrest in the G1 phase through the downregulation of cyclin D1 and CDK-2 genes exploring the anticancer potential of AgNPs and selenium SeNPs nanoparticles green-synthesized with berberine (Ber) in a human hepatocellular carcinoma cell line (HepG2) [55].

Unlike natural or biologically derived genoprotectants, Argovit AgNPs, a synthetic formulation, offer the advantage of consistent composition and stability, making them a reliable candidate for therapeutic applications. The present study contributes to this body of knowledge by investigating the genoprotective effects of Argovit against cytosine arabinoside, which is an antineoplastic drug.

Various publications were devoted to studying vivo genoprotective effects against cyclophosphamide-induced damage. The Mammalian Erythrocyte Micronucleus Test guidelines from the OECD recommend using cyclophosphamide as a negative control for genotoxicity studies [30]. Hence, our group’s present and recent study [14] revealed that AgNPs hold great potential as antineoplastic genoprotectants. AgNPs present a new class of antineoplastic genoprotectants with the advantage of a controlled composition. According to our best knowledge, antineoplastic genoprotectors known before our works were plant extracts, the composition of which is difficult to control. It should also be considered that AgNP antineoplastic genoprotectors have other advantages as can be seen from the analysis presented in the Supplementary Information as Table S1, which summarizes the work of several authors on the genoprotective effect of different compounds against antineoplastic agents and UV radiation [14,49,56,57,58,59,60,61,62,63,64,65,66,67]. Our research results have shown that AgNPs, precisely the Argovit^TM^ formulation, exhibit high activity and selectivity against cancer tumor cells, leaving healthy cells unharmed [11]. Furthermore, the Argovit^TM^ formulation lacks genotoxic effects in human lymphocytes [19] and is environmentally harmless [13]. The benefits of Argovit^TM^ AgNPs listed above make this formulation a promising candidate for cancer therapy.

This work shows the need for further experiments. (1) The genoprotective effect of AgNPs was revealed for Argovit™ formulation. It is necessary to investigate if this effect is observed only for this specific AgNP formulation or others. (2) This work was coducted using healthy mice. The following study should be carried out on mice with cancer. (3) Shedding light on the mechanism of the genoprotector phenomenon revealed for AgNPs is also pending. All these studies will permit us to evaluate the perspectives on applying the AgNP genoprotective effect in modern clinical practice.

## 5. Conclusions

The present research showed the efficacy of AgNPs in reducing the harm to genetic material caused by an alkylating agent, which is a first-line chemotherapy medication Ara-C. After administering three doses of Ara-C, the number of micronuclei in immature and accumulated erythrocytes increased by three and two times, respectively. However, when three doses of AgNPs (6 mg/kg each) were given after three doses of Ara-C (6 mg/kg each), the number of micronuclei in immature and accumulated erythrocytes decreased by 3.72 and 2 times, respectively. It was revealed that AgNPs could be considered a new class of promising synthetic antineoplastic genoprotectants, along with well-known organic compounds obtained from plants, microorganisms, and mollusks.

The findings of this study demonstrate the potential of AgNPs as a genoprotective agent against Ara-C-induced genotoxicity, significantly reducing the frequency of micronucleated erythrocytes. While the phenotypic outcomes confirm the protective effects, the underlying molecular mechanisms remain to be elucidated. Future investigations focusing on vital regulatory pathways, such as CDK1/2 expression and other markers of DNA repair and cell cycle regulation, will be crucial to understanding the precise mechanisms of action. This study provides a foundation for further exploration of AgNPs not only as genoprotectants but also as potential modulators of cellular responses to DNA damage, paving the way for their integration into safer therapeutic strategies against cancer-induced genotoxicity.

## Figures and Tables

**Table 1:** The average weight of mice in studied groups.

Group	Compound (Hours)	Average Weight the First Day (g)	Average Weight on the Last Day (g)	Difference (%)
1	Water (0, 24, 48)	20.19 ± 2.23	21.4 ± 2.53	4.27
2	Ara-C (0)	18.83 ± 3.21	20.01 ± 2.56	6.27
3	Ara-C (0, 24, 48)	16.70 ± 2.42	17.17 ± 1.27	2.78
4	AgNPs (0, 24, 48)	20.69 ± 2.86	21.89 ± 3.31	5.84
5	Ara-C (0) + AgNPs (24, 48, 72)	22.15 ± 2.16	21.33 ± 2.44	−3.70
6	Ara-C (0, 24, 48) + AgNPs (72, 96, 120)	19.43 ± 2	23.22 ± 2.4	19.49
7	Ara-C (0, 48, 96) + AgNPs (24, 72, 120)	21.38 ± 3.27	21.34 ± 3.72	−0.18

## Data Availability

Dataset available on request from the authors.

## Supplementary Materials

The following supporting information can be downloaded at https://www.mdpi.com/article/10.3390/ijerph21121689/s1, Figure S1: Supplementary data1 Excel Sheet_(ANOVA and Tukey), Table S1: In vivo genoprotective effect of different compounds towards cyclophosphamide and other antineoplastic agents and UV radiation. Ref. [68] is cited in Supplementary Materials.
