# Supplementary material for: Protective Effect of Silver Nanoparticles Against Cytosine Arabinoside Genotoxicity: An In Vivo Micronucleus Assay
Source: Int J Environ Res Public Health. 2024 Dec 18;21(12):1689. doi: 10.3390/ijerph21121689 (PMC11675496; doi:10.3390/ijerph21121689)
Supplement: Supplementary file 1 [file ijerph-21-01689-s001.zip › ijerph-3326407-supplementary.pdf]

Table Analyzed	MNE /10 000 TE Mean, S, N					
Two-way ANOVA	Ordinary					
Alpha	0.05					
Source of Variation	% of total variation	P value	P value summary	Significant?		
Hours	10	<0.0001	****	Yes		
Group	5.3	<0.0001	****	Yes		
ANOVA table	SS (Type III)	DF	MS	F (DFn, DFd)	P value	
Hours	19453	7	2779	F (7, 616) = 11	P<0.0001	
Group	10035	6	1673	F (6, 616) = 6.4	P<0.0001	
Residual	161376	616	262			
Data summary						
Number of columns (Group)	7					
Number of rows (Hours)	8					
Number of values	630					

Table Analyzed	PCE /1000 TE Mean, S, N,				
Two-way ANOVA	Ordinary				
Alpha	0.05				
Source of Variation	% of total variation	P value	P value summary	Significant?	
Hours	4.1	0.0001	***	Yes	
Group	1.4	0.1188	ns	No	
ANOVA table	SS (Type III)	DF	MS	F (DFn, DFd)	P value
Hours	297389	7	42484	F (7, 698) = 4.3	P=0.0001
Group	100036	6	16673	F (6, 698) = 1.7	P=0.1188
Residual	6853733	698	9819		
Data summary					
Number of columns (Group)	7				
Number of rows (Hours)	8				
Number of values	712				

Table Analyzed	MN PCE /1000 TE Mean, S, N,				
Two-way ANOVA	Ordinary				
Alpha	0.05				
Source of Variation	% of total variation	P value	P value summary	Significant?	
Hours	12	<0.0001	****	Yes	
Group	3.9	0.0001	***	Yes	
ANOVA table	SS (Type III)	DF	MS	F (DFn, DFd)	P value
Hours	672	7	96	F (7, 616) = 12	P<0.0001
Group	223	6	37	F (6, 616) = 4.7	P=0.0001
Residual	4864	616	7.9		
Data summary					
Number of columns (Group)	7				
Number of rows (Hours)	8				
Number of values	630				

Figure S1: Supplementary data1 Excel Sheet_(ANOVA and Tukey)

Table S1: In vivo genoprotective effect of different compounds towards cyclophosphamide and other antineoplastic agents and UV radiation

No.	Characteristics of genoprotectant	Experiment details of antineoplastic agent application	Ref.

	Compound	Genoprotectant	Dose and route of administration	Antineoplastic agent, dose, and administration route	Biological model	Application scheme	Observed genoprotective effect	
1	AgNPs coated with PVP	AgNPs	6 mg/kg, oral	CP, 50 mg/kg, intraperitoneal injection	Murine BALB/, peripheral blood	Post-treatment effect: CP was administered for 1 day and then AgNPs for 3 days.<br><br>Alternate treatment: CP (0, 48, 96 h) + AgNPs (24, 72, 120 h)	Post-treatment: 2.0-time decrease of micronuclei in reticulocytes and 1.32-time decrease in accumulated erythrocytes<br><br>Alternate treatment: 2.0-time decrease in micronuclei in reticulocytes and 1.76-time decrease in accumulated erythrocytes	[1] our group
2	Isoflavone	Isoflavone semi	1.71, 8.54,	CP, 50 mg/kg,	Swiss albino	Pre-treatment:	Pre-treatme	[2]

	obtained from soybean (plant)	synthetic derived from daizain	and 42.85 mg/kg, oral	intraperitoneal injection	mouse, bone marrow micronucleus assay	mice received isoflavone for 14 days and CP was administered on the last day<br><br>Simultaneous treatment: isoflavone and CP were administrated once at the same time<br><br>Post-treatment: isoflavone was administered 24 h after the CP administration (1 time)	nt: 5.32, 2.83, and 2.44-fold decrease in reticulocyte micronuclei, respectively, compared to the effect with CP alone<br><br>Simultaneous treatment: 1.05, 1.38, and 1.06-time decrease in micronuclei in reticulocytes compared to CP alone.<br><br>Post-treatment: 2.61, 1.13, and 1.29-time reduction of micronuclei in

						reticulocytes compared with CP alone	
3	Triterpenoid saponin obtained from plant	SFGC	4.5, 9, and 45 mg/kg, oral	CP, 20 mg/kg, intraperitoneal injection	Swiss albino mice, aberrations in somatic cells of the bone marrow	Pre-treatment: SFGC was administered 1 day before 1 dose of CP<br><br>Post-treatment: 1 dose of SFGC was administered 1 day after CP administration	Pre-treatment: chromosomal aberrations decreased by 1.42, 1.72, and 2.05 times<br><br>Post-treatment: 1.27, 1.46, and 1.85-time decrease in the chromosomal aberrations	[3]
4	Plant-derived open-chain flavonoid	Chalcone	25 and 50 mg/kg, oral	CP, 50 mg/kg intraperitoneal injection	Swiss Webster mouse bone marrow micronucleus assay and comet assay	Simultaneous treatment: one dose of chalcone with one dose of cyclophosphamide<br><br>Pre-treatment: for 5 consecutive days Chalcona	Simultaneous treatment: the generation of micronuclei in reticulocytes decreased by 1.6 and 1.72 times.	[4]

						was administrated, on the last day, 2 h later one dose of CP<br><br>Post-treatment: 1 dose of CP, 6 or 12 h later chalcone was administrated	Pre-treatment: the generation of micronuclei in reticulocytes decreased by 2 and 2.51 times<br><br>Post-treatment: the generation of micronuclei in reticulocytes decreased by 0.99 and 1.01 times	
5	Water soluble tannin extracted from a plant (Lafoensia pacari)	Punicalagin	12.5, 25, and 50 mg/kg, intraperitoneal injection<br><br>12.5 and 25 mg/kg<br><br>Increased from 12.5 to 25 mg/kg	CP, 50 mg/kg, intraperitoneal injection	Swiss Webster mice, one marrow micronucleus assay, and comet assay	Simultaneous treatment: genoprotectant (each dose) was administered together with a 50 mg/kg single dose intraperitoneal injection of CP<br><br>Pre-treatment: intraperitoneal injection of punicalagin	Simultaneous treatment: the number of micronuclei generated in reticulocytes decreased by 1.46, 2.125, and 2.72 times	[5]

						for 5 days, on the last day CP was administered<br><br>Post-treatment: CP injection and 6 and 12 h later pulicalagin was administrated (12.5 and 25 mg/kg doses, respectively)	Pre-treatment: the generation of micronuclei in reticulocytes decreased by 2.38 and 3.58 times<br><br>Post-treatment: 8.5-time decrease in the generation of micronuclei in reticulocytes.	
6	Hydrolysable tannin extracted from a plant	Gemin D	25, 50, and 100 mg/kg, intraperitoneal injection 25 and 50 mg/kg<br><br>Gradually increasing from 25 to 50 mg/kg	CP, 50 mg/kg intraperitoneal injection	Swiss Webster mouse, bone marrow micronucleus assay	Simultaneous treatment: a single dose of Gemin D together with CP<br><br>Pre-treatment: for 5 days Gemin D was injected, two hours after the last injection CP was	Simultaneous treatment: the generation of micronuclei in reticulocytes decreased by 1.4, 1.7, and 2.34 times.<br><br>Pre-treatment: the	[6]

						administered<br><br>Post-treatment: first CP was administered and 6 h later 25 mg/kg dose was injected and 12 h later 50 mg/kg dose was injected	generation of micronuclei in reticulocytes decreased by 1.91 and 2.95 times<br><br>Post-treatment: the generation of micronuclei in reticulocytes decreased by 0.99 times	
7	Compound obtained from bacteria Saccharomyces cerevisiae	Carboxymethylglucan K2 and K3	50 mg/kg, intraperitoneal and intravenous injection	CP, 80 mg/kg, intraperitoneal injection	Swiss mouse, micronucleus assay in bone marrow erythrocytes	Pre-treatment: Carboxymethylglucan K2 and K3 were injected 24 h before the injection with CP	Intravenous K2 and K3: 1.58 and 2.57 times decreased, respectively, the genotoxic damage in reticulocytes. Intraperitoneal K2 and K3: decreas	[7]

							ed genotoxic damage in reticulocytes by 1.52 and 5.56 times, respectively.	
8	Plant extract	Salvia officinalis extract	50, 100, and 150 mg/kg, oral	CP, 40 mg/kg, intraperitoneal injection	Wistar rats, micronucleus assay in bone marrow erythrocytes	Pre-treatment: 7 days of treatment with extract, CP was administered post-treatment on the 7th day	The generation of micronuclei in reticulocytes decreased by 1, 1.80, and 2.27 times	[8]
9	Plant extract	Rubus imperialis extract	50, 250, and 500 mg/kg, oral	CP, 50 mg/kg, intraperitoneal injection	Swiss albino mice, Micronucleus assay in bone marrow erythrocytes and comet assay	Simultaneous treatment: single dose application of both at the same time	The generation of micronuclei in reticulocytes decreased by 5.43, 10.33 and 10.33 times. At concentrations of 250 and 500 mg/kg of extract alone, a clastogenic and aneugeni	[9]

							c effect was reported, as the frequency of micronuclei in bone marrow reticulocytes increased.	
10	Oyster polysaccharides	Oyster polysaccharides	100 and 200 mg/kg, oral	CP, 50 mg/kg, intraperitoneal injection	BALB/c mice, micronucleus assay in bone marrow erythrocytes	Pre-treatment: 7 days of treatment with genoprotectant, CP administered post-treatment at 7th day	The generation of micronuclei in reticulocytes decreased by 1.10 and 2.1 times	[10]
11	Isolate from a fungus Aspergillus niger	Carboxymethyl-chitin–glucan (CMCG)	100 and 200 mg/kg, intraperitoneal or oral	CP, 80 mg/kg, two intraperitoneal injections 24 hours apart	ICR mouse, micronucleus assay in bone marrow erythrocytes	Pre-treatment: Intraperitoneal: the two different doses of CMCG were administered before CP<br><br>Oral: two different doses of CMCG were administered before CP	Intraperitoneal: the generation of micronuclei in reticulocytes decreases by 2 and 2.3 times, respectively.<br><br>Oral: the generation of micronuclei in	[11]

							reticulocytes decreases by 1.74 and 2 times, respectively.	
12	Plant extract (Punica granatum)	Plant extract Punica granatum (PLE)	400, 600, and 800 mg/kg, oral	CP, 40 mg/kg, intraperitoneal injection	Swiss albino mice, Micronucleus assay in bone marrow erythrocytes	Pre-treatment 7 consecutive days of PLE with different doses, respectively, and on the last day, two hours, CP was administered after the PLE.	The genotoxic damage decreased by 1.5, 1.6, and 2.27 times, respectively	[12]
13	Plant juice	Diplotaxis tenuifolia	1,000 1,400, and 2,000 mg/kg, oral	CP, 50 mg/kg, post-treatment intraperitoneal injection	Swiss female and male mice, micronucleus assay in bone marrow erythrocytes	Pre-treatment 15 days, CP was administered on the 15th day	The generation of micronuclei in reticulocytes decreased by1.38, 1.66 and 1.78	[13]
14	Silver nanoparticles	AgNPs	6 mg/kg, oral	Ara-C, (50 mg/kg), intraperitoneal injection	BALB/c, micronucleus assay in peripherally blood	Best post-treatment effect: Ara-C was applied 3 days and then 3 days of AgNPs	Micronuclei in reticulocytes and in accumulated erythrocytes decreased by 3.72 and	Present work

							2 times, respectively	
15	Silver nanoparticles	AgNPs coated with glycyrrhizic acid, from Liquorice root (SN-GLY)	50 mg/kg (administration route is not mentioned)	Exposition to UV radiation	Swiss albino mouse micronucleus assay in peripheral blood erythrocytes	Application of SN-GLY just after being exposed to 4 Gy gamma radiation.	The generation of micronuclei in reticulocytes at 24 and 48 hours after radiation decreased 4.6 and 3.6 times, respectively.	[14]
16	Compound extracted from grape	Naringin	50, 250, y 500 mg/kg. oral	Ifosfamide (60 mg/kg), oral	NIH mice micronucleus assay in peripheral blood erythrocytes	Simultaneous treatment: The doses were administered for 4 days respectively, 1 h later Ifosfamide was administered	Decreased 1.66, 1.84, and 2.18 times, it could not decrease the cytotoxic damage at 48 hours and in the maximum concentration, on the contrary, it increased once the cytotoxic	[15]

	damage produced by naringin

[1] Castañeda-Yslas IY, Torres-Bugarín O, García-Ramos JC, Toledano-Magaña Y, Radilla-Chávez P, Bogdanchikova N, et al. AgNPs Argovit™ modulates cyclophosphamide-induced genotoxicity on peripheral blood erythrocytes in vivo. Nanomaterials 2021;11:2096. https://doi.org/10.3390/nano11082096.

[2] Delarmelina JM, Dutra JCV, Batitucci M do CP. Antimutagenic activity of ipriflavone against the DNA-damage induced by cyclophosphamide in mice. Food and Chemical Toxicology 2014;65:140–6. https://doi.org/10.1016/j.fct.2013.12.028.

[3] Melek FR, Aly FA, Kassem IAA, Abo-Zeid MAM, Farghaly AA, Hassan ZM. Three further triterpenoid saponins from Gleditsia caspica fruits and protective effect of the total saponin fraction on cyclophosphamide-induced genotoxicity in mice. Zeitschrift Fur Naturforschung - Section C Journal of Biosciences 2015;70:31–7. https://doi.org/10.1515/znc-2014-4132.

[4] Da Silva Lima DC, Do Vale CR, Veéras JH, Bernardes A, Peérez CN, Chen-Chen L. Absence of genotoxic effects of the chalcone (E)-1-(2-hydroxyphenyl)-3-(4-methylphenyl)-prop-2-en-1-one) and its potential chemoprevention against DNA damage using in vitro and in vivo assays. PLoS One 2017;12. https://doi.org/10.1371/journal.pone.0171224.

[5] Carneiro CC, da Costa Santos S, de Souza Lino R, Bara MTF, Chaibub BA, de Melo Reis PR, et al. Chemopreventive effect and angiogenic activity of punicalagin isolated from leaves of Lafoensia pacari A. St.-Hil. Toxicol Appl Pharmacol 2016;310:1–8. https://doi.org/10.1016/j.taap.2016.08.015.

[6] Carneiro CC, de Moraes-Filho AV, Fernandes AS, da Costa Santos S, de Melo e Silva D, Chen LC. Cytotoxic and Chemopreventive Effects of Gemin D Against Different Mutagens Using In Vitro and In Vivo Assays. Anticancer Agents Med Chem 2017;17:712–8. https://doi.org/10.2174/1871520616666160906092502.

[7] Chorvatovičová D, Navarová J. Suppressing effects of glucan on micronuclei induced by cyclophosphamide in mice. Mutation Research Letters 1992;282. https://doi.org/10.1016/0165-7992(92)90088-Y.

[8] Üstün Alkan F, Esen Gürsel FE, Ateş A, Özyürek M, Güçlü K, Altun M. Protective effects of Salvia officinalis extract against cyclophosphamide-induced genotoxicity and oxidative stress in rats. Turk J Vet Anim Sci 2012;36:646–54. https://doi.org/10.3906/vet-1105-36.

[9] Alves ABCR, Santos RS Dos, Calil SDS, Niero R, Lopes JDS, Perazzo FF, et al. Genotoxic assessment of Rubus imperialis (Rosaceae) extract in vivo and its potential chemoprevention against cyclophosphamide-induced DNA damage. J Ethnopharmacol 2014;153:694–700. https://doi.org/10.1016/j.jep.2014.03.033.

[10] Lin S, Hao G, Long M, Lai F, Li Q, Xiong Y, et al. Oyster (Ostrea plicatula Gmelin) polysaccharides intervention ameliorates cyclophosphamide—Induced genotoxicity and hepatotoxicity in mice via the Nrf2—ARE pathway. Biomedicine and Pharmacotherapy 2017;95:1067–71. https://doi.org/10.1016/j.biopha.2017.08.058.

[11] Chorvatovicova D, Machova E, Sandula J. Ultrasonication: the way to achieve antimutagenic effect of carboxymethyl-chitin-glucan by oral administration. MUTATION RESEARCH-GENETIC TOXICOLOGY AND ENVIRONMENTAL MUTAGENESIS 1998;412:83–9. https://doi.org/10.1016/S1383-5718(97)00176-9.

[12] Dassprakash MV, Arun R, Abraham SK, Premkumar K. In vitro and in vivo evaluation of antioxidant and antigenotoxic potential of Punica granatum leaf extract. Pharm Biol 2012;50. https://doi.org/10.3109/13880209.2012.689771.

[13] López Nigro MM, Peroni RN, Ayllón-Cabrera I, Schiariti Lampropulos VE, Roma MI, Carballo MA. In vivo antigenotoxic activity of Diplotaxis tenuifolia against cyclophosphamide-induced DNA damage: Relevance of modulation of hepatic ABC efflux transporters. Mutat Res Genet Toxicol Environ Mutagen 2018;836:72–8. https://doi.org/10.1016/j.mrgentox.2018.06.006.

[14] Chandrasekharan DK, Nair CKK. Effect of silver nanoparticle and glycyrrhizic acid (SN-GLY) complex on repair of whole body radiation-induced cellular DNA damage and genomic instability in mice. Int J Low Radiat 2010;7. https://doi.org/10.1504/IJLR.2010.037668.

[15] Alvarez-González I, Madrigal-Bujaidar E, Dorado V, Espinosa-Aguirre JJ. Inhibitory effect of naringin on the micronuclei induced by ifosfamide in mouse, and evaluation of its modulatory effect on the Cyp3a subfamily. Mutation Research - Fundamental and Molecular Mechanisms of Mutagenesis 2001;480–481. https://doi.org/10.1016/S0027-5107(01)00197-X.
